# Eye-Tracking the Production of Counterfactual Alternatives to Narrative Events in Autistic Adults

**DOI:** 10.1177/17470218261419775

**Published:** 2026-01-23

**Authors:** Heather J. Ferguson, Marchella Smith

**Affiliations:** 1 School of Psychology, Keynes College, University of Kent, Canterbury, UK

**Keywords:** autism, counterfactuals, language production, eye-tracking, social cognition

## Abstract

Research has found atypical counterfactual production in autistic children, yet intact counterfactual reasoning in autistic adults. To date, however, no research has investigated counterfactual production in autistic adults. The current study combined a counterfactual production task with eye-tracking in the visual world paradigm to examine how autistic and neurotypical adults plan and initiate counterfactual responses. Autistic participants showed slower speech initiation and more disfluencies, consistent with broader speech production patterns, but did not experience greater difficulties with counterfactual production itself. Both groups also showed a comparable preference to produce additive counterfactuals, suggesting developmental convergence by adulthood. By contrast, while both groups showed a strong preference to produce and fixate human causes, this bias was stronger among neurotypical adults than autistic participants, who more often considered physical causes and were less influenced by salient social cues. Additionally, speech-locked eye movements revealed group differences: neurotypical adults flexibly modulated their gaze to human causes depending on whether they produced factual or counterfactual responses, whereas autistic adults showed more uniform gaze patterns across conditions. These findings highlight shared competencies but distinct social-cognitive strategies in counterfactual production across groups.

## Introduction

Counterfactual thinking can be prompted in tasks that present a narrative event (Guajardo & Turley-Ames, 2004), for example: ‘Jack gives Lola some money and sends her off to buy a balloon. Jack warns Lola to watch out for the prickly chestnut tree on the way. After she has bought the balloon, Lola excitedly runs towards Jack to show it to him, not paying attention to what is around her. She runs too close to the tree and her balloon catches on a branch and bursts’, then asks participants to describe *what could have happened differently so that the balloon did not burst*. Appropriate responses to this question should depict what would have been true under alternative hypothetical circumstances (e.g. ‘If Lola had not run so close to the tree, then her balloon would not have caught on the branch and burst’). Counterfactual thinking has been observed in children as young as 3 years of age (Harris et al., 1996; Leevers & Harris, 2000; Riggs et al., 1998) and is thought to help regulate emotions, such as regret and relief. For example, it allows us to learn from our own or other people’s previous mistakes by generating/planning multiple alternative options for how to approach similar scenarios in the future (Begeer et al., 2009; Epstude & Roese, 2008).

The dual mental models account (Byrne, 2002; Byrne & Tasso, 1999; McConnell-Ginet & Fauconnier, 1987; Santamaría et al., 2005) proposes that counterfactuals are cognitively demanding because they trigger two incompatible representations (e.g. Santamaría et al., 2005; see also Gómez-Veiga et al., 2010). Producing a counterfactual alternative to events requires people to (a) understand the fictitious hypothetical event (that Lola did not run close to the tree, so her balloon did not get caught on the branch and burst), to subsequently, (b) recall the implied factual event (that Lola ran close to the tree, so her balloon got caught on the branch and burst) and understand that only the latter is true in reality (Byrne, 2002; Fauconnier, 1987). Experimental work has shown that the maintenance and switching between these two imagined worlds incurs increased processing costs (see Ferguson et al., 2019; Kulakova & Nieuwland, 2016 for reviews). Moreover, successful counterfactual thinking engages the same set of executive functions (e.g. working memory, inhibitory control and cognitive flexibility) that mediate successful understanding of others’ mental states (i.e. theory of mind [ToM]; Drayton et al., 2011; Riggs et al., 1998; Van Hoeck et al., 2014; but see Perner et al., 2004), and it has been proposed that development of counterfactual thinking may be a necessary but not sufficient mechanism to develop explicit perspective-taking ability (Ferguson et al., 2019; Kulakova & Nieuwland, 2016). In this paper, we explore counterfactual production in autistic adults, who are characterised by atypical social communication and executive functioning, and show different patterns of developing counterfactual thinking in childhood (e.g. Morsanyi & Handley, 2012; Scott & Baron-Cohen, 1996).

### Counterfactual Thinking in Autism

Autistic children (aged 9–13 years) have been shown to produce fewer correct counterfactual alternatives compared to chronological age-, IQ- and gender-matched neurotypical children (Grant et al., 2004) and are less able to use counterfactuals to understand others’ emotions (Begeer et al., 2014). These patterns have been explained in terms of impaired meta-learning in autism (Frith & Happé, 1994; van Boxtel & Lu, 2013; Van de Cruys et al., 2014), which disrupts the ability to contextualise incoming information and make predictions based on previous experience.

However, other research has suggested that the ability to generate counterfactuals is comparable between autistic and neurotypical children, though the reasoning strategy may be distinct. In a cross-sectional design, Begeer et al. (2009) found that at 6- to 8-year-old autistic children generated fewer *subtractive* counterfactuals (e.g. ‘If only I had not done. . .’), but a similar number of *additive* counterfactuals (‘If only I had done’) compared to age-matched neurotypical children. By 10- to 12-year-old autistic children, this pattern reversed such that autistic children generated fewer *additive* counterfactuals but a similar number of *subtractive* counterfactuals as neurotypical children. This suggests that counterfactual thinking develops through distinct strategies in autistic and neurotypical people. Moreover, the delayed development of additive counterfactual thinking may reflect the greater imagination, creativity and flexibility that are required to generate new adaptive response options to approach the scenario (Begeer et al., 2009), all of which are thought to be atypical in autism (Black et al., 2018; Smith et al., 2024).

More recent research has explored how counterfactuals are understood in real time, including in autistic adults, using sensitive online research techniques (e.g. eye-tracking and event-related potentials [ERPs]). This work has shown that people process counterfactual information in real time and can rapidly imagine a hypothetical counterfactual version of the world, even when this violates real-world constraints (e.g. Ferguson, 2012; Ferguson & Cane, 2015; Ferguson & Sanford, 2008; Ferguson et al., 2008, 2010; Nieuwland, 2013; Nieuwland & Martin, 2012). Importantly, this work suggests that online counterfactual understanding is undiminished or even enhanced among autistic adults compared to neurotypical adults (Black et al., 2018, 2019; Ferguson et al., 2019, 2021; Wimmer & Ferguson, 2025). For example, when participants were eye-tracked while reading short factual and counterfactual narratives, autistic adults detected anomalies – characterised by longer reading times and increased regressions – in a similar, or even faster time course than neurotypical adults (Black et al., 2018, 2019; Ferguson et al., 2019). Similarly, ERPs recorded while participants read scenarios that set up a factual or counterfactual world, then either maintained the counterfactual world or switched back to the factual world, showed that autistic and neurotypical adults elicited indistinguishable neural responses to contextually inconsistent words, despite group-level differences in ToM and cognitive flexibility (Ferguson et al., 2022).

These patterns of intact counterfactual understanding in adults contrast with the difficulties seen among children (Begeer et al., 2009; Grant et al., 2004; Leevers & Harris, 2000), perhaps reflecting an extended developmental trajectory for counterfactual thinking in autism (i.e. reaching the same level of performance/strategy, but at a later stage of development compared to neurotypical people). It is also possible that the modality of counterfactual thinking affects group-level performance. Research in adults has focused primarily on counterfactual *comprehension*, whereas research in children’s work has focused on the *production* of counterfactual thoughts, meaning that very little is known about counterfactual production in autistic adults. Thus, it might be that counterfactual understanding is intact in autism, but distinct strategies and difficulties emerge when autistic adults are required to express counterfactual alternatives into words.

### The Current Study

The current study investigated the production of counterfactual alternatives to narrative events in autistic and neurotypical adults, and used eye-tracking to capture real-time strategies of planning and initiating a response. The production task was presented within a visual world paradigm (Allopenna et al., 1998; Cooper, 1974) in which participants first listened to a narrative event then viewed a scene depicting these events while they responded to factual (e.g. ‘what happened that caused the balloon to burst?’) and counterfactual (e.g. ‘what could have happened differently so that the balloon did not burst?’) questions. Eye movements were recorded during visual scene presentation and time-locked to the onset of verbal response production. This allowed us to assess both the content of counterfactual production responses in autistic versus neurotypical adults and the real-time attentional strategies that they employed to plan/initiate these responses.

While a large body of research has examined eye movements during language comprehension (for reviews, see Huettig et al., 2011; Ito, 2024), work combining eye movements with language production is much more limited (Griffin, 2004). Nevertheless, research using this approach has shown that visual attention plays a crucial role in language planning, as speakers attend more to objects that are meaningful for their speech goals (as opposed to visually salient; Henderson et al., 2018; Rehrig et al., 2020) and plan their speech sequentially by fixating on objects before they mention them in speech (Griffin & Bock, 2000). Cognitive effort also influences visual attention during language production as speakers look longer at objects when the cognitive effort of retrieving the word and constructing an utterance is high (Lee et al., 2024; Meyer & Dobel, 2003).

Research suggests that basic visual attention around scenes follows distinct patterns in autistic and neurotypical groups (Au-Yeung et al., 2015, 2018; Benson & Fletcher-Watson, 2011; Benson et al., 2012), and these differences are particularly pronounced for social objects (Benson et al., 2016; Fletcher-Watson et al., 2009). Neurotypical people tend to preferentially fixate on social stimuli in a scene (e.g. people, faces, eyes) rather than on low-level salient features (Gliga & Csibra, 2007). This implicit social bias might reflect attempts to understand other people’s mental states (e.g. ToM) and could enhance success in generating causal explanations for unusual events. By contrast, autistic people have a relatively diminished preference for looking at social stimuli (e.g. Kirchner et al., 2011; Klin et al., 2002; Riby et al., 2013; see Chita-Tegmark, 2016, for meta-analysis), and the size of this effect correlates with autism symptom severity (Bird et al., 2011; Chawarska et al., 2013). For example, in a recent study, Barzy et al. (2020) used eye-tracking glasses to measure social attention during a face-to-face conversation, and found that autistic adults spent less time looking at socially relevant objects in the scene (i.e. experimenter’s face) and more time looking at non-social information compared to neurotypical adults; this reduced social bias in autism increased when the task became more difficult (see also Freeth & Bugembe, 2019). Reduced social gaze is interpreted as a means to manage the high cognitive load of managing a conversation and understanding others’ mental states (Doherty-Sneddon & Phelps, 2005; Doherty-Sneddon et al., 2002, 2013).

In line with research on counterfactual production in autistic children (Begeer et al., 2009; Grant et al., 2004; Leevers & Harris, 2000), we tested the hypothesis that autistic adults would show atypical counterfactual production compared with neurotypical adults. Specifically, we expected autistic adults to show greater difficulty in responding to the questions (i.e. slower latency to fluent speech onset and increased number of disfluencies) and tested whether this difficulty would be more pronounced when producing counterfactual compared to factual responses (e.g. Ferguson et al., 2019). In addition, we expected autistic adults to show a reduced preference for producing additive counterfactuals compared to neurotypical adults (as has been reported among children; Begeer et al., 2009) and a diminished frequency of producing human relative to physical cause alternatives than neurotypical adults due to group-level differences in preference for social/non-social stimuli (e.g. Barzy et al., 2020; Kirchner et al., 2011; Klin et al., 2002; Riby et al., 2013). In eye movement data, we hypothesised that the different preferences for social/non-social stimuli would be evident in overall reduced and delayed gaze preference towards human causes among autistic adults compared to neurotypical peers. In addition, we expected that the difficulty with counterfactual production in autism would be evident as overall reduced fixations on the human cause when producing counterfactual versus factual alternatives, as they allocate attention more widely around the scene (rather than directing attention to appropriate causal factors).

## Method

### Participants

Twenty-four autistic adults and 24 neurotypical adults were recruited from the Autism Research at Kent database and through local advertising. All participants were aged over 18 years old, were native English speakers and had full-scale IQ scores greater than 70. Participant groups were statistically matched on age, gender and Intelligence Quotient^1^ (IQ, Wechsler Adult Intelligence Scale, WAIS-III or WAIS-IV; Wechsler, 1997, 2008), but were not matched on autism spectrum quotient (AQ; Baron-Cohen et al., 2001b; see Table 1).

**Table 1.** Demographic Information for Participants in the Autistic and Neurotypical Groups; *M* (*standard deviation*). IQ Was Determined Using Wechsler Adult Intelligence Scale (Scaled Scores).

Measure	Autistic (*N* = 24)	Neurotypical (*N* = 24)	*t*-value	*p*-value
Gender (m:f)	17:7	17:7		
Age (years)	33.84 (10.74)	33.58 (11.96)	0.08	.936
Verbal IQ	105.58 (12.19)	100.50 (8.08)	1.70	.095
Performance IQ	111.58 (19.79)	103.21 (12.47)	1.75	.086
Full scale IQ	109.08 (15.24)	102.21 (9.56)	1.87	.068
Total AQ score	31.25 (8.44)	17.63 (6.85)	6.14	<.001
ADOS-2 module 4	8.08 (4.82)	—	*—*	—

*Note.* ADOS = autism diagnostic observation schedule; AQ = autism spectrum quotient.

All autistic participants had a formal diagnosis of an autism spectrum condition from a trained clinician (5 autistic disorder, 5 autism spectrum disorder, 13 Asperger’s syndrome, 1 pervasive developmental disorder not otherwise specified). In addition, current autistic symptomology was assessed in this group by a research-reliable, trained researcher using module 4 of the autism diagnostic observation schedule (ADOS-2; Lord et al., 2000, see Table 1). Videos were double coded by an additional trained, research-reliable researcher, to ensure reliability of scoring (inter-rater reliability was found to be excellent with intraclass correlation of .89). Twelve of the 24 autistic participants met the clinical ADOS cut-off (i.e. a score of 7 or above), indicating significant autistic traits.^2^ Neurotypical participants did not report any developmental or psychiatric diagnoses.

Ethical approval for this study was received from the University of Kent School of Psychology Ethics Committee. Participants provided written informed consent and were fully debriefed following completion of the experiment. They received £10 per hour for their participation, plus additional travel expenses.

### Materials

Twenty stories depicting mildly negative events were developed from those used by Guajardo and Turley-Ames (2004) and adjusted to be suitable for adults. Each story was manipulated such that the negative event had both a physical, uncontrollable cause (e.g. the weather) and a human, controllable cause (the person’s decision or action). Stories differed from Guajardo and Turley-Ames (2004) in that the stories were written in the third person rather than the second.

Each image/story was paired with two question prompts, one factual and one counterfactual. Questions in the factual condition always began, ‘What happened that caused. . .’, and counterfactual questions began, ‘What could have happened differently so that. . .’. An example story and corresponding questions are shown below:

Jack is taking his niece Lola out for a day at the fairground. Lola sees someone selling colourful balloons and asks if Jack will buy her one. Jack gives her some money and sends her off to buy herself a balloon. Jack warns Lola to watch out for the prickly chestnut tree on the way. After she has bought the balloon, Lola excitedly runs towards Jack to show it to him, not paying attention to what is around her. She runs too close to the tree and her balloon catches on a branch and bursts.

Factual question: *What happened that caused the balloon to burst?*
Counterfactual question: *What could have happened differently so that the balloon did not burst?*

One version of each item was assigned to 1 of 2 presentation lists, with each list containing 20 unique experimental items, 10 in each of the 2 conditions. Participants were randomly assigned to one of these two lists, which ensured that across these lists (and therefore across participants) each image/story was seen in both question prompt conditions. The full list of images, stories and questions can be found on the OSF repository at https://osf.io/a7bqs/.

### Procedure

Participants completed the study in a quiet laboratory at the University of Kent. Eye movements from the right eye (viewing was binocular) were tracked using a desk-mounted EyeLink 1000 plus eye tracker in remote tracking mode (SR Research Ltd.), running at 500 Hz sampling rate. Participants were seated 60 cm from the eye-tracking camera and 70 cm from the computer screen. At the beginning of the experiment, and as required thereafter, the eye-tracker was calibrated and validated against nine fixation points, using the standard EyeLink calibration procedure. This procedure took about half a minute, and the entire session lasted for about 25 min.

**Figure 1.**
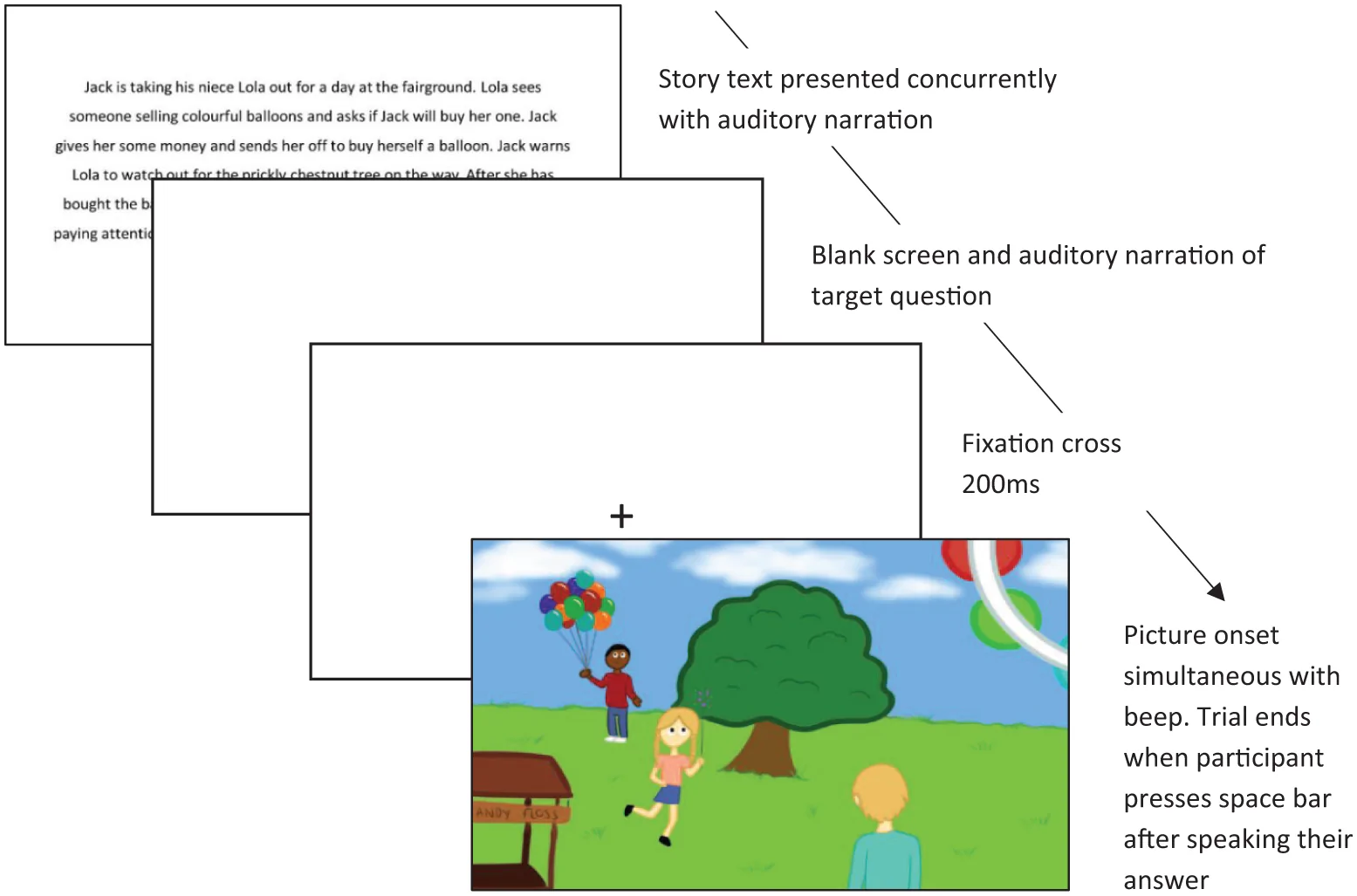
Example of an experimental trial sequence.

The experiment was controlled using Experiment Builder software, and the experimental procedure is illustrated in Figure 1. Participants were told they would listen to short stories, then be asked to verbally respond to a question after each story. Full instructions were presented onscreen and explained verbally, and participants were invited to ask any questions before the experiment began. Participants completed 2 practice trials to ensure they understood the procedure, before completing the 20 experimental trials in a randomised order. Each trial began with a drift correction check (central fixation point on the screen). Following successful fixation on this point, the story text and associated audio were presented simultaneously. Text was displayed for the length of the audio, after which a 1,000 ms blank screen was shown. The question prompt was then presented auditorily while the screen remained blank, followed by a 200 ms central fixation cross. The onset of the trial image was time-locked to an auditory beep (to indicate that participants could begin their verbal response), and images stayed onscreen for a maximum of 30,000 ms, or until the participant pressed the spacebar to indicate they had finished their answer.

Auditory recordings of the stories and question prompts were recorded by a female, native English speaker who spoke with a neutral tone. Auditory files were presented as 22 kHz stereo sound clips via headphones connected to the eye-tracker PC, with a microphone attached for speech recording. Story text was presented on screen in black, size 26, Calibri font on a white background. Images depicting the outcome described in the story were drawn in a cartoon style by an artist, and clearly depicting the physical and human causes within each story, as well as the consequence. They were presented to participants on a 24-inch colour monitor in 1,024 × 768 pixels resolution.

## Results

The datasets and transcripts of verbal responses, as well as analysis scripts, are available on the OSF project page at https://osf.io/a7bqs/.

### Speech Production Measures

The task employed a mixed design, crossing the between-subjects factor Group (autistic vs. neurotypical) and within-subjects factor Question type (factual vs. counterfactual). Speech production data were analysed on five key measures for the first cause produced on each item. First, we calculated the *latency to fluent speech onset* (in msec), as the time from image/beep onset to the start of fluent speech (excluding disfluencies and hesitations). Second, the *number of speech disfluencies* was calculated as the total number of disfluencies from image/beep onset to the end of the first causal response. Third, the preference to produce *human versus physical causes* was calculated as the proportion of trials on which participants produced a human cause (e.g. ‘Lola should have been paying more attention’) versus physical cause (e.g. ‘the prickly spikes from the chestnut tree burst her balloon’). Fourth, the preference to produce *additive versus subtractive counterfactual causes* was calculated as the proportion of trials on which participants produced an additive cause (e.g. ‘if she’d been watching out’) versus subtractive cause (e.g. ‘if Lola had not run so close to the tree’) in response to a counterfactual question. Finally, *word order* was coded as the word number in a sentence that participants first mentioned the human (only for trials on which they produced a human cause^3^). Coding for these last three measures was conducted by two independent raters who were blind to participant group and condition, and responses were presented in random order. Where disagreements arose between raters, coding decisions were resolved by consensus to ensure consistent application of the coding scheme. In a very small number of items, both human and physical causes were mentioned; in these cases, the first mentioned cause was coded. Data for each measure, group and condition are summarised in Table 2.

**Table 2.** Descriptive Statistics (Mean and Standard Deviations in Parenthesis) for Each Speech Production Measure, Group and Condition.

Measure	Autistic		Neurotypical	
	Counterfactual	Factual	Counterfactual	Factual
Latency to fluent speech onset (ms)	2,262 (2,238)	1,927 (1,464)	1,769 (1,453)	1,500 (775)
Number of speech disfluencies	0.31 (0.46)	0.31 (0.46)	0.15 (0.36)	0.17 (0.38)
Human versus physical causes	0.92 (0.27)	0.55 (0.50)	0.98 (0.14)	0.52 (0.50)
Additive versus subtractive counterfactual causes	0.78 (0.41)	—	0.82 (0.39)	—
Word order (first mention of human cause)	1.13 (0.37)	1.06 (0.36)	1.09 (0.32)	1.11 (0.41)

Data for *latency to fluent speech onset, number of speech disfluencies* and *human versus physical causes* were analysed using a glmer model (using the lme4 R package), crossing group (autistic vs. neurotypical) and condition (factual vs. counterfactual) as fixed factors and with random effects for participants and items. Data on *additive versus subtractive counterfactual causes were analysed* using a glmer model with group (autistic vs. neurotypical) as the fixed factor and random effects for participants and items. Data for *word order* were analysed using a lmer model, crossing group (autistic vs. neurotypical) and condition (factual vs. counterfactual) as fixed factors and with random effects for participants (the model did not converge with a random effect for items as this contributed zero variance). Initial models specified the maximal random structure supported by the design, with condition as a random slope by participant, and condition × group as random slopes by item. If a model failed to converge, we used a backward fitting approach to simplify the random structure (Barr et al., 2013). Final model structures are detailed in the analysis script on OSF.

#### Latency to Fluent Speech Onset

Analysis revealed that participants were faster to initiate fluent speech in response to a factual than counterfactual question (1,712 ms vs. 2,012 ms; β = −229.87, SE = 85.12, *t* = 2.70, *p* = .007), and that neurotypical adults were faster to initiate fluent speech than autistic adults (1,634 ms vs. 2,092 ms; β = −378.60, SE = 176.88, *t* = 2.14, *p* = .032). The group × condition interaction was not significant, β = 57.53, SE = 159.73, *t* = 0.36, *p* = .719.

#### Number of Speech Disfluencies

Analysis revealed that autistic adults made more speech disfluencies than neurotypical adults (0.31 vs. 0.16; β = −1.04, SE = 0.40, *z* = 2.60, *p* = .009). There was no significant effect of condition, β = .06, SE = 0.18, *z* = 0.32, *p* = .752, and no condition × group interaction, β = .18, SE = 0.35, *z* = 0.52, *p* = .602.

#### Human Versus Physical Causes

Analysis revealed that participants produced a higher proportion of human causes when answering a counterfactual than factual question (0.95 vs. 0.53; β = −3.11, SE = 0.28, *z* = −11.27, *p* < .001). A Wilcoxon signed rank test with a chance set at .5 showed that participants were significantly biased to produce a human cause (i.e. above chance set at .5) for counterfactual responses, *V* = 105,728, *p* < .001, but were equally likely to produce a human or physical cause for factual responses, *V* = 60,579, *p* = .143. In addition, neurotypical adults had a stronger preference to produce human causes than autistic adults (0.75 vs. 0.73; β = .63, SE = 0.29, *z* = 2.18, *p* = .029). Furthermore, the group × condition interaction was significant, β = 1.51, SE = 0.55, *z* = 2.74, *p* = .006, showing that the group difference in human cause preference was only significant when participants were answering a counterfactual question (β = −1.38, SE = 0.52, *z* = −2.64, *p* = .008), and not when answering a factual question (β = .13, SE = 0.21, *z* = 0.62, *p* = .533).

#### Additive Versus Subtractive Counterfactual Causes

Overall, participants showed a significant bias to produce an additive cause (tested using a Wilcoxon signed rank test with chance set at .5; *V* = 87,938, *p* < .001). The preference to produce an additive versus subtractive cause in response to a counterfactual question did not differ by group, β = .27, SE = 0.41, *z* = 0.65, *p* = .515.

#### Word Order

The order of mentioning the human cause in a verbal response did not differ by group (β = −.01, SE = 0.04, *t* = 0.04, *p* = .972) or condition (β = −.02, SE = 0.04, *t* = 0.59, *p* = .579), and the interaction was non-significant (β = .09, SE = 0.08, *t* = 1.14, *p* = .260). The mean word orders reported in Table 2 show that participants tended to mention the human first in the sentence.

### Eye Movements at Image Onset

Two areas of interest (AOIs) were defined in each experimental image: the human cause (the person who could have caused the negative outcome, for example, Lola because she was not paying attention and walked too close to the tree with her balloon) and the physical cause (the object that could have caused the negative outcome, for example, the tree that burst the balloon). AOIs did not overlap.

Analyses examined global eye movement behaviour towards these two AOIs, time-locked to the onset of the target image. Four measures were identified for analysis. *First fixation onset* is the time at which participants first fixated each AOI, which reflects the speed of initial attentional capture. *First fixation duration* captures the length of this first fixation on each AOI, providing an index of early attentional engagement. The *proportion of fixations* on each AOI before speech onset indicates relative allocation of visual attention across competing stimulus types, and the sum *gaze duration* on each AOI prior to speech onset reflects the total time spent looking at each AOI during this pre-speech window, offering a measure of sustained attention. Given the significant imbalance of human versus physical cause responses across groups and conditions, these analyses included response type as an additional variable. Data for each measure, group and condition are summarised in Table 3.

**Table 3.** Descriptive Statistics (Mean and Standard Deviations in Parenthesis) for Each Eye Movement Measure, Group and Condition.

Measure	Autistic				Neurotypical			
	Counterfactual		Factual		Counterfactual		Factual	
	Human AOI	Physical AOI	Human AOI	Physical AOI	Human AOI	Physical AOI	Human AOI	Physical AOI
First fixation onset (ms)								
Human cause response	1,178 (1,941)	2,328 (2,862)	1,101 (2,349)	2,215 (2,627)	1,137 (1,584)	1,900 (1,728)	1,217 (1,691)	1,736 (1,491)
Physical cause response	1,612 (2,808)	1,199 (984)	1,143 (1,492)	1,586 (1,878)	1,390 (1,252)	852 (426)	1,543 (1,857)	1,106 (1,155)
First fixation duration (ms)								
Human cause response	227 (158)	260 (171)	241 (130)	250 (138)	277 (175)	274 (194)	291 (152)	263 (175)
Physical cause response	239 (189)	187 (93)	256 (130)	281 (234)	303 (204)	268 (176)	297 (238)	267 (204)
Proportion pre-speech fixations								
Human cause response	0.30 (0.27)	0.14 (0.20)	0.33 (0.32)	0.15 (0.22)	0.29 (0.32)	0.12 (0.19)	0.29 (0.32)	0.11 (0.19)
Physical cause response	0.22 (0.27)	0.18 (0.19)	0.25 (0.29)	0.19 (0.23)	0.22 (0.30)	0.24 (0.18)	0.25 (0.31)	0.25 (0.27)
Pre-speech gaze duration (ms)								
Human cause response	590 (669)	288 (441)	650 (640)	363 (607)	524 (554)	259 (412)	485 (420)	217 (353)
Physical cause response	465 (456)	553 (884)	375 (438)	314 (379)	253 (343)	291 (241)	326 (388)	330 (358)

*Note.* AOI = area of interest.

We note that previous research has highlighted a potential issue with directly comparing the proportion of fixations or the sum gaze duration on two AOIs in the same image (Heller et al., 2008), since time spent looking at one AOI necessarily reduces available time for the other. Our paradigm reduces this limitation because the AOIs do not dominate the visual scene, and participants spent the majority (~60%) of their time looking elsewhere. This means that the impact of looking at one AOI at any time has a much less direct impact on the other AOI than studies that use simple or binary displays, and reduces the concern that the two AOIs are directly competing for the same limited pool of attentional time. In addition, as a language production study, eye movements are not influenced by bottom-up cues from external language input (as they are in language comprehension studies), which can dominate gaze. Consequently, the AOI-based analyses we report are appropriate and robust for this production task.

Data were analysed using glmer models (Shapiro-Wilk tests were significant for all measures, *p* < .001), crossing group (autistic vs. neurotypical), condition (factual vs. counterfactual), AOI (human vs. physical) and response type (human vs. physical) as fixed factors and with random effects for participants and items, and maximal random slopes (see the analysis script on OSF for final model structures).

#### First Fixation Onset

Participants were faster to first fixate an AOI when they later produced a physical cause response compared to a human cause response (1,351 ms vs. 1,589 ms; β = 264, SE = 107, *t* = 2.47, *p* = .014). Additionally, response type interacted with AOI (β = 1,056, SE = 216, *t* = 4.90, *p* < .001), showing a significant human versus physical AOI bias when participants gave a human cause response (β = −773, SE = 299, *z* = 2.59, *p* = .010), but not when they gave a physical cause response (β = 282, SE = 343, *z* = 0.823, *p* = .411). An interaction between AOI and group (β = −664, SE = 229, *t* = 2.91, *p* < .01) showed that autistic adults were faster to first fixate the physical AOI than neurotypical adults (β = 415, SE = 163, *z* = 2,54, *p* = .011), but the time to first fixate the human AOI did not differ between groups (β = −249, SE = 150, *z* = 1.66, *p* = .096). None of the other effects reached significance (*t*s < 1.33, *p*s > .182).

#### First Fixation Duration

Autistic adults showed shorter first fixation durations compared to neurotypical adults (248 ms vs. 278 ms; β = 36.86, SE = 18.02, *t* = 2.05, *p* = .041). None of the other effects reached significance (*t*s < 1.71, *p*s > .088).

#### Proportion of Pre-Speech Fixations

Analysis showed that participants were overall more likely to fixate the human AOI than the physical AOI (0.28 vs. 0.15; β = −.07, SE = 0.03, *t* = 2.27, *p* = .023). This AOI effect interacted with response type (β = −.17, SE = 0.04, *t* = 4.65, *p* < .001), such that the human AOI fixation preference was only significant when participants gave a human cause response (β = .16, SE = 0.03, *z* = 5.34, *p* < .001), and not when they later gave a physical cause response (β = −.01, SE = 0.04, *z* = 0.25, *p* = .802). None of the other effects reached significance (*t*s < 1.94, *p*s > .052).

#### Pre-Speech Gaze Duration

None of the effects reached significance (*t*s < 1.33, *p*s > .185).

### Eye Movements Around Speech Onset

**Figure 2.**
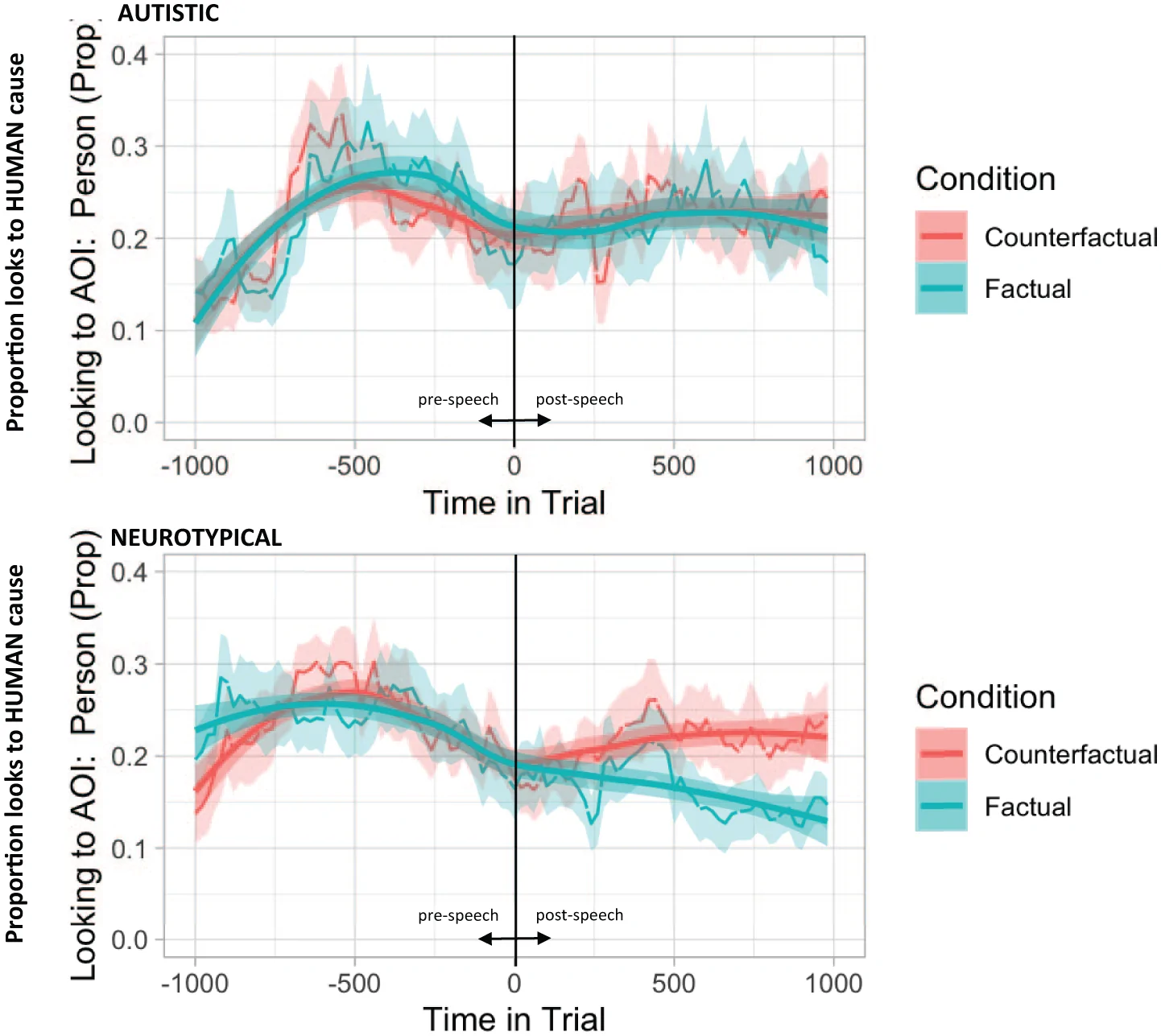
Time course of fixations towards the human cause AOI over the pre-speech onset and post-speech onset periods, when participants produced a human cause response, in autistic (top panel) and neurotypical (bottom panel) groups for counterfactual and factual conditions. *Note.* AOI = area of interest.

To examine temporal effects, eye movement data were analysed in two time periods time-locked to the onset of fluent speech (calculated for each participant and trial): pre-speech onset (i.e. fixations in the 1,000 ms before speech onset, reflecting planning of speech) and post-speech onset (i.e. fixations in the 1,000 ms after speech onset, reflecting initiation of speech). Fixations were broken into 20 ms time bins, and the spatial coordinates were mapped onto AOIs as a function of time. Given the significant differences in producing a human cause between conditions and groups, and that observers are known to look longer at objects they will subsequently mention (Griffin, 2004; Griffin & Bock, 2000; Henderson et al., 2018; Rehrig et al., 2020), this analysis controlled for content differences by focusing exclusively on trials in which participants produced a human cause (74% of trials overall) and examined the proportion of looks to the human cause AOI over time. This enabled us to examine whether the time-course of planning a human causal response differs across conditions and groups when the speech content was comparable. Visual preferences to the human cause AOI were represented by a binary term in each 20 ms time bin, where ‘1’ indicated a fixation on the human AOI and ‘0’ indicated no fixation. The proportion of fixations to the human cause AOI is plotted for each group and condition in Figure 2, from 1,000 ms before speech onset to 1,000 ms after speech onset.

Data were analysed separately for each time period using generalised mixed models and growth curve analysis (Mirman et al., 2008), using the ‘lme4’ and ‘eyetrackingR’ packages in RStudio (R Core Team). Fitting growth curve models to the data to test different shapes of visual bias over time allows us to capture the effects of group and condition as the verbal description was being planned/initiated, while also testing for variance between and within individuals. In this study, third-degree orthogonal polynomials, incorporating intercept, linear and quadratic components, were used to model the time course of anticipatory bias over the 1,000 ms pre-speech onset period and 1,000 ms post-speech onset period (see Mirman et al., 2008). Final models included contrast-coded fixed effects for group and condition (−0.5 vs. 0.5) alongside the time polynomials, random effects of participants and items (both with random slopes for condition and the time polynomials). Statistical effects are presented in Table 4.

**Table 4.** Statistical Results From the Growth Curve Analyses Examining Fixations Towards the Human Cause AOI Over the Pre-Speech Onset and Post-Speech Onset Periods, When Participants Produced a Human Cause Response. Ot1 and ot2 Refer to Linear and Quadratic Models of Time, Respectively.

Statistical effect	Looks to Human Cause AOIs					
	Pre-Speech Onset			Post-Speech Onset		
	Est.	SE	*z*-value	Est.	SE	*z*-value
Group	−0.158	0.239	−0.661	0.322	0.368	0.876
Condition	0.086	0.388	0.222	0.060	0.347	0.172
ot1	0.710	0.974	0.466	0.168	0.813	0.206
ot2	−1.575	0.609	−2.589**	−0.317	0.675	−0.469
Group: condition	0.058	0.458	0.127	0.476	0.532	0.89
Group: ot1	1.367	1.073	1.273	0.119	1.327	0.089
Group: ot2	−1.463	0.814	−1.798	−0.532	0.907	−0.586
Condition: ot1	−0.223	0.291	−0.768	−0.842	0.287	−2.938**
Condition: ot2	0.221	0.284	0.781	−0.009	0.280	−0.033
Group: condition: ot1	1.028	0.571	1.801	2.192	0.564	3.882***
Group: condition: ot2	−1.530	0.559	−2.738**	−0.646	0.551	−1.172

*Note.* AOI = area of interest.

**p < .01. ***p < .001.

#### Pre-Speech Onset Looks to Human Causes

Analyses revealed a significant effect of quadratic time, reflecting a general increase, then a decrease in fixations on the human cause AOI over the 1,000 ms pre-speech onset period. There was also a significant interaction between group × condition × quadratic time. To explore this three-way interaction, post hoc analyses were conducted separately for each group. Analyses revealed that in the neurotypical group, the condition × quadratic fit interaction was significant (β = .95, SE = 0.34, *z* = 2.76, *p* = .006), reflecting a shallower curvature – increase then decrease – in looks to the human cause AOI when planning a human response in the factual condition, and a later but steeper rise and fall in the counterfactual condition. By contrast, the condition × quadratic fit interaction was not significant in the autistic group (β = −.42, SE = 0.42, *z* = 1.00, *p* = .312), suggesting that autistic participants showed comparable patterns of fixations to the human cause AOI when planning a factual and counterfactual human cause response.

#### Post-Speech Onset Looks to Human Causes

Analyses revealed significant interactions between condition × linear time and group × condition × linear time. To explore this three-way interaction, post hoc analyses were conducted separately for each group. In the neurotypical group, the group × linear fit interaction was significant (β = −2.43, SE = 0.38, *z* = 6.45, *p* < .001), reflecting increasing looks to the human cause AOI when participants were initiating a counterfactual description and decreasing looks to the human cause AOI when participants were initiating a factual description. The group × linear fit interaction was not significant in the autistic group (β = .08, SE = 0.42, *z* = 0.18, *p* = .856).

## Discussion

The current study combined a counterfactual production task with eye-tracking in the visual world paradigm (Allopenna et al., 1998; Cooper, 1974) to capture the production of counterfactual alternatives to narrative events and the real-time strategies that autistic and neurotypical adults use to plan and initiate these responses. We tested the prediction that autistic adults, like autistic children, would show atypical counterfactual production compared with neurotypical adults (i.e. slower latency to fluent speech onset and increased number of disfluencies). We also expected autistic adults to show distinct counterfactual production strategies, including a diminished frequency of producing human cause alternatives and a reduced preference for producing additive counterfactuals compared to neurotypical adults. In eye movement data, we hypothesised that autistic adults would show overall reduced and delayed gaze preference towards human causes compared to neurotypical peers, and reduced fixations on the human cause when producing counterfactual versus factual alternatives (i.e. reflecting weaker specificity in causal attributions).

### Production Responses and Strategies

Analysis of production responses revealed that autistic adults were overall slower to initiate a verbal response and made more disfluencies prior to fluent speech onset compared to neurotypical adults, likely reflecting general group-level differences in language production processes and a tendency to produce more disfluent speech in autism (Engelhardt et al., 2017; Pirinen et al., 2023, 2024). All participants were slower to initiate a counterfactual response than a factual one, supporting the proposal that producing a counterfactual alternative to events involves additional processing steps (e.g. to inhibit the factual world and imagine an alternative) and is cognitively demanding (Byrne, 2002; Byrne & Tasso, 1999). Importantly, this counterfactual delay was no greater among autistic compared to neurotypical adults, suggesting that autistic adults did not experience a specific or heightened difficulty with counterfactual language production.

Analysis of preferences to produce human versus physical causes revealed subtly different strategies among autistic and neurotypical adults. While both groups showed a clear preference to produce human causes over physical causes, this bias was stronger among neurotypical than autistic participants, especially in the counterfactual condition (98% vs. 92%, respectively). This pattern reflects a generally stronger bias to attend to social information among neurotypical people (Gliga & Csibra, 2007) and a *relatively* diminished – but still evident – preference for social stimuli among autistic people (e.g. Kirchner et al., 2011; Klin et al., 2002; Riby et al., 2013). The finding that group-level differences in human-cause preference were only present when producing counterfactual, but not factual explanations, suggests that thinking counterfactually may incur additional cognitive demands to understand other people’s mental states and generate alternative causal explanations for unusual events. For example, generating human counterfactual alternatives may involve considering the protagonist’s actions and intentions, meaning that it may draw more strongly on perspective-taking and mentalising (ToM), which are typically diminished in autism (Baron-Cohen et al., 2001a; Deschrijver et al., 2016; Gökçen et al., 2016; Laghi et al., 2016; Peterson et al., 2009; Scheeren et al., 2013).

In contrast to the group-level strategies on producing human versus physical causes, the overall preference to produce additive versus subtractive counterfactual causes did not differ between groups. This finding contrasts with previous research that has shown distinct patterns of developing additive versus subtractive counterfactual production strategies among autistic and neurotypical children. Begeer et al. (2009) found that the production of additive counterfactuals increased from 6 to 12 years old in neurotypical children, but remained stable across this age range in autistic children; at 10 to 12 years old, autistic children generated fewer additive counterfactuals than neurotypical children. Findings from the current study, therefore, provide novel evidence that autistic people experience an acceleration in additive counterfactual production between the ages of 12 and young adulthood (this strategy shift is delayed in autism compared to neurotypical development), meaning that additive versus subtractive counterfactual reasoning strategies are comparable between autistic and neurotypical people by the time they reach young adulthood.

Atypical additive counterfactual reasoning in autistic children has been explained in terms of the greater load it places (relative to subtractive counterfactual reasoning) on underlying mechanisms of imagination and cognitive flexibility (Begeer et al., 2009), both of which are known to be diminished in autistic children (Baron-Cohen, 1987; Lewis & Boucher, 1988; Wing & Gould, 1979; Wolfberg et al., 2012). However, group-level differences in imagination and cognitive flexibility persist into adulthood (e.g. Black et al., 2018; Ferguson et al., 2022; Smith et al., 2024), despite displaying comparable strategies for additive counterfactual production. As such, the current study suggests that alternative mechanisms support the development of additive counterfactual thinking in addition to/instead of imagination and cognitive flexibility. Further research is needed to identify these mechanisms and the age at which they trigger a change in counterfactual strategies from middle childhood to young adulthood.

It is important to acknowledge that the current task differed from Begeer et al. (2009) in key ways that may have supported better performance in our participants. The current study provided participants with a visual scene of described events, while Begeer et al.’s (2009) study presented short stories in text-only format, meaning that children needed to generate and hold the scene in mind themselves (i.e. scene construction; Hassabis et al., 2007). Scene construction ability is known to be diminished in autism; autistic adults produce less detailed and coherent scene construction descriptions than neurotypical adults (Black et al., 2018; Smith et al., 2024). However, when autistic people are provided with visual cues about what information to provide, they produce comparable scene construction descriptions as neurotypical people (Anger et al., 2019). Therefore, it is possible that the visual world format used in the current study scaffolded scene construction ability in a way that reduced load on imagination and executive functions and enabled the generation of novel/imaginative (i.e. additive) counterfactual alternatives. Indeed, Leevers and Harris (2000) found that giving an instruction to ‘imagine’ described events improves counterfactual thinking in neurotypical children, but impairs it in autistic children. When a visual scene is not available to support scene construction and clarify the communicator’s intentions, autistic people may experience difficulty engaging their imagination to produce additive counterfactual alternatives, and instead are more likely to adopt a logical reasoning response bias (Ferguson et al., 2019; Leevers & Harris, 2000).

### Eye Movements During Language Production

Analysis of eye movements examined both early attentional biases at image onset (prior to speech) and the temporal dynamics of fixations to the human cause around speech onset. At image onset, participants showed a rapid bias towards social information, with earlier fixations and a higher proportion of pre-speech looks to the human versus the physical cause. Contrary to our prediction for delayed and reduced social orienting in autism, first fixations on the human cause were equally fast in autistic and neurotypical groups, and autistic adults even showed a stronger human versus physical first fixation bias. Around speech onset, there were no overall group differences in fixations to the human cause during either planning (pre-speech) or initiation (post-speech) of a human cause response. These findings suggest that autistic adults, like neurotypical peers, prioritise social information to support narrative comprehension at the level of visual attention. Thus, a dissociation emerges between early attentional biases and later production choices: while autistic adults display spontaneous early prioritisation of social content, this translates into a robust linguistic preference for human causes that is slightly reduced compared to neurotypical adults.

Eye movements were also sensitive to the type of verbal response being produced, but these patterns differed across groups. In the pre-speech period (i.e. the 1,000 ms directly before fluent speech onset, reflecting planning), neurotypical adults distinguished between factual and counterfactual human cause responses, showing earlier and more stable fixations on the human cause AOI in the factual condition and a later, steeper shift in the counterfactual condition. By contrast, autistic adults did not show condition-related differences, instead allocating attention to the human cause in a similar way when planning both factual and counterfactual human cause responses. In the post-speech onset period (i.e. the 1,000 ms following fluent speech onset, reflecting initiation), neurotypical adults again showed condition-sensitive gaze, with fixations to the human cause increasing during counterfactual responses and decreasing during factual responses, while autistic adults did not show such differentiation. Because analyses were restricted to trials on which participants produced a human cause response, these group effects cannot be explained by differences in speech content or mention order. Rather, they suggest that neurotypical adults flexibly modulate their attention to socially relevant information depending on the type of causal reasoning required, whereas autistic adults engage more uniformly with the scene, showing less condition-specific adjustment in gaze during both speech planning and initiation.

Reduced sensitivity to condition in the autistic group may indicate tighter coupling between gaze and ongoing speech planning, such that visual attention reflects immediate production demands rather than broader social-causal considerations (Griffin & Bock, 2000). Notably, group differences in fixations emerged primarily in the factual condition, and were broadly similar between groups in the counterfactual condition. Thus, it may be that producing factual explanations exerts lower cognitive and linguistic demands, which frees up resources to enable neurotypical participants to attend to socially informative features of the scene. Autistic participants, by contrast, may rely on a more stable event-based, visuospatial strategy across conditions, which makes the group differences more noticeable when the task is less demanding (Barzy et al., 2020; Doherty-Sneddon & Phelps, 2005; Doherty-Sneddon et al., 2002; Kirchner et al., 2011; Klin et al., 2002; Riby et al., 2013; see Chita-Tegmark, 2016). To our knowledge, this study is the first to examine speech-locked eye movements in autistic adults using the visual-world paradigm, and the results point to meaningful group differences in how visual attention is integrated with factual and counterfactual reasoning during language production.

## Conclusions

In sum, the current experiment revealed both similarities and differences in counterfactual language production and gaze strategies between autistic and neurotypical adults. Autistic participants initiated speech more slowly and with more disfluencies, consistent with broader speech production patterns (Mody & Belliveau, 2012), but did not show specific difficulties with counterfactual production. This suggests that counterfactual ability remains intact in adulthood, in line with prior evidence of undiminished or enhanced counterfactual comprehension (Black et al., 2018, 2019; Ferguson et al., 2019). Autistic adults also showed comparable use of additive counterfactual strategies, suggesting that the distinct patterns observed in childhood reflect a more protracted developmental trajectory than in neurotypical peers (Begeer et al., 2009, 2014; Grant et al., 2004). However, subtle group differences emerged in attributional strategies: while both groups consistently preferred human causes when producing counterfactual alternatives, this bias was stronger among neurotypical than autistic adults, who more often considered physical causes and were less influenced by salient social cues. In addition, speech-locked eye movements revealed that only neurotypical adults modulated their attention to human causes differently in factual versus counterfactual conditions, both during speech planning and initiation, whereas autistic adults showed more uniform gaze patterns across conditions. Together, these findings highlight the complex interplay of social, cognitive and visual strategies in counterfactual production in autism and underscore the need for future research on how visual cues might scaffold earlier development of counterfactual thinking in autistic children.

## Notes

1. Note that IQ scores showed a trend between groups (*p*s < .1), with autistic participants scoring higher on average compared to typically developing participants.

2. We note that while the ADOS total score is widely used as a ‘gold standard’ measure of ASC severity, it is influenced by chronological age, language aptitude and co-occurring clinical conditions (Gotham et al., 2009).

3. Note that on 13% of human cause responses, participants did not explicitly mention the human (e.g. ‘[she] should have been more careful’). In these cases, no word order value was coded.
